# The Brief Case: *Vibrio cholerae* gastroenteritis but not cholera

**DOI:** 10.1128/jcm.00602-26

**Published:** 2026-11-11

**Authors:** Claire Duployez, Frédéric Wallet, Rémi Le Guern, Fanny Lemonnier, Noémie Curame, Romain Hund, Blandine Huyghe, Caroline Rouard, Caroline Loïez

**Affiliations:** 1 Service de Bactériologie, CHU Lille, Lille, France; 2 Univ. Lille, Lille, France; 3 Service de Néphrologie, CHU Lille, Lille, France; 4 Centre National de Référence Vibrions et Choléra, Institut Pasteur 27058, Paris, France; Endeavor Health, Evanston, Illinois, USA

**Keywords:** *Vibrio cholerae*, gastroenteritis, non-O1/non-O139, bacteriological techniques, syndromic PCR, atpA gene

## CASE

A 54-year-old French woman with polycystic kidney disease on peritoneal dialysis following renal transplant rejection presented to the emergency department with a 10-day history of watery diarrhea. She had no fever, no vomiting, and no additional symptoms except mild abdominal pain. She described watery stools and a feeling of dehydration with 1 kg weight loss over 48 h. She had lived permanently in France and reported no recent travel abroad or seafood consumption. No family members reported similar symptoms. Physical examination revealed stable vital signs (blood pressure of 146/111 mmHg, heart rate of 86 bpm, and temperature of 36.8°C) and a soft, non-tender abdomen without organomegaly or peritoneal signs. Laboratory investigations showed a normal white blood cell count (6,910 cells/mm^3^; reference range 4,000–10,000 cells/mm^3^), C-reactive protein 9 mg/L (reference range <6 mg/L), potassium 5.4 mmol/L (reference range 3.4–4.4 mmol/L), chloride 93 mmol/L (reference range 98–107 mmol/L), and mild hyponatremia (129 mmol/L; reference range 136–145 mmol/L). Renal function parameters were consistent with chronic kidney disease (creatinine 86 mg/L; reference range 5–8 mg/L, blood urea nitrogen 1.29 g/L; reference range 0.15–0.5 g/L).

**Fig 1:**
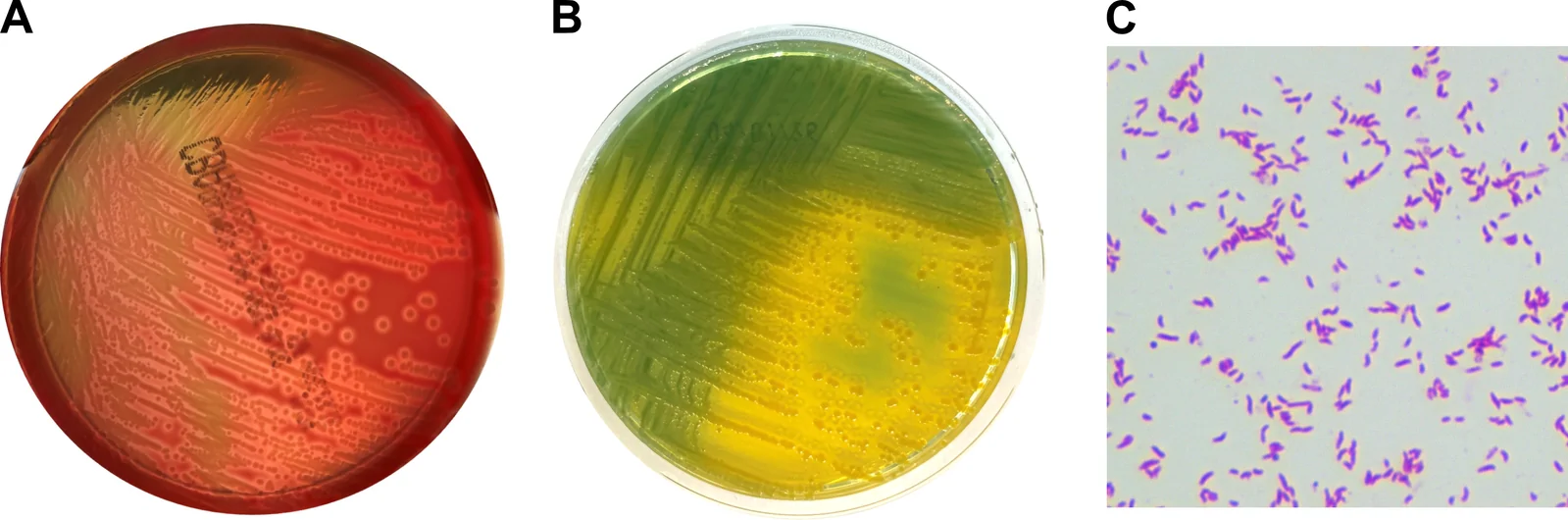
(**A**) β-hemolytic colonies on blood agar following 6 h of incubation at 35°C–37°C. (**B**) Yellow colonies on TCBS agar following 6 h of incubation at 35°C–37°C. (**C**) Gram stain showing curved and straight gram-negative bacilli (×1,000).

Stool specimens were tested using the BD MAX Enteric Bacterial Panel and Extended Enteric Bacterial Panel (Becton Dickinson, Le Pont-de-Claix, France): the *atp*A gene (encoding the α-subunit of bacterial ATP synthase) (1) was detected, indicating presumptive *Vibrio* species (including *V. cholerae*, *V. parahaemolyticus,* and *V. vulnificus*). Culture on blood agar and thiosulfate-citrate-bile salts-sucrose (TCBS) within 6 h yielded, respectively, β-hemolytic colonies and yellow colonies (sucrose +) (Fig. 1A and B). MALDI-TOF mass spectrometry (MALDI Biotyper, Bruker Daltonics, Wissembourg, France) identified the organism as *V. cholerae* with a high-confidence score of 2.45, resolving the interpretive ambiguity raised by the molecular panel result.

Given the patient’s chronic renal disease and immunosuppressed state, empiric treatment with azithromycin was initiated on the basis of the preliminary *Vibrio* species identification, and the patient experienced rapid clinical improvement with resolution of diarrhea within 48 h. Antimicrobial susceptibility testing (disk diffusion, EUCAST/CA-SFM 2025 breakpoints) confirmed susceptibility to cephalosporins, tetracyclines, fluoroquinolones, trimethoprim-sulfamethoxazole, and azithromycin.

The isolate was referred to the National Reference Center for Vibrios and Cholera (Institut Pasteur, Paris, France), where identification was confirmed by a specific PCR amplification of the intergenic spacer region (between the 16S and 23S rDNAs) (2). Slide agglutination with O1 and O139 antisera was negative, and PCR for the *ctx*A gene (encoding the subunit A of the cholera toxin) was negative. The isolate was therefore classified as *V. cholerae* non-O1/non-O139, lacking cholera toxin genes, consistent with non-cholera *V. cholerae*. Whole-genome sequence (Illumina, San Diego, USA) of the strain confirmed species identification (rMLST) and absence of *ctx*A gene. Virulence genes *mak*A, *rtx*A, and *nan*H were detected. The source of infection was not identified despite a thorough clinical history.

## DISCUSSION

*Vibrio cholerae* belongs to the family *Vibrionaceae*, with more than 150 species, primarily inhabiting coastal and estuarine waters. *Vibrios* are motile, gram-negative (Fig. 1C), oxidase-positive bacilli. Among *Vibrio* species pathogenic to humans, two clinically important groups are recognized: (i) toxigenic *V. cholerae* O1 and O139 strains, responsible for cholera, and (ii) non-cholera vibrios, including *V. cholerae* non-O1/non-O139, as well as other species such as *V. parahaemolyticus* and *V. vulnificus*.

These three species differ in their clinical profiles and epidemiology, and distinguishing among them matters for both patient management and public health response. *V. parahaemolyticus* is the most common cause of *Vibrio*-associated gastroenteritis worldwide, typically linked to raw or undercooked seafood consumption and generally self-limiting in immunocompetent hosts. *V. vulnificus*, while less frequent, is associated with high-case fatality rates in patients with liver disease or immunosuppression. *V. cholerae* non-O1/non-O139 strains occupy an intermediate clinical position: most infections manifest as self-limited gastroenteritis, but invasive disease, including bacteremia, has been reported in patients with underlying comorbidities (3–7). More details about *Vibrio* species are presented in Table 1.

**TABLE 1:** Main *Vibrio* strains involved in human pathology

*Vibrio* species	*V. cholerae* toxigenic O1 and O139	Non-O1 non-O139 (and non-toxigenic O1) *V. cholerae*	*V. mimicus*	*V. parahaemolyticus*	*V. fluvialis*	*V. alginolyticus*	*V. vulnificus*
Common properties	- Family: *Vibrionaceae*; genus: *Vibrio* - Gram appearance: small, straight, slightly curved, curved, or comma-shaped gram-negative rods - Growth characteristics: facultative anaerobe, oxidase +, catalase +, motile, H_2_S– - Distribution: primarily aquatic species, marine and estuarine environments						
Contamination mode	Consumption of contaminated food or water, or person-to-person transmission (fecal-oral route)	Consumption of contaminated food or water Exposure to contaminated water		Consumption of raw or undercooked contaminated seafood Exposure to seawater			
Cholera toxin	Yes	Sometimes	Sometimes	No	No	No	No
Disease presentation	Cholera	Gastroenteritis, ear and wound infections, rarely septicemia	Gastroenteritis	Gastroenteritis, wound infections, rarely septicemia	Gastroenteritis	Ear and wound infections	Gastroenteritis, wound infection, septicemia
Appearance on TCBS	Yellow (sucrose +)	Yellow (sucrose +)	Green (sucrose −)	Green (sucrose −)	Yellow (sucrose +)	Yellow (sucrose +)	Green (sucrose −) rarely yellow
Appearance on blood agar	β-hemolytic	β-hemolytic	β-hemolytic	β-hemolytic or nonhemolytic	β-hemolytic	nonhemolytic, can swarm	β-hemolytic
NaCl requirement	Nonhalophilic	Nonhalophilic	Nonhalophilic	Halophilic	Halophilic	Halophilic	Halophilic

More than 200 serogroups of *V. cholerae* have been described, and only O1 and O139 are associated with cholera when the strain produces the cholera toxin. The isolation of a strain belonging to the species *V. cholerae* is therefore not sufficient to confirm a case of cholera in non-endemic areas, and clinicians and microbiologists must resist the reflexive association between the species name and the disease. While travel to endemic regions and raw seafood consumption remain the most commonly identified risk factors for *Vibrio* gastroenteritis in Europe, the absence of these exposures during the patient’s interview should not exclude the diagnosis. Immunosuppression, chronic kidney disease, chronic liver disease, and diabetes are recognized host risk factors for severe non-cholera infections. This case illustrates both points: the patient lacked classical exposure history yet developed symptomatic infection attributable to her immunosuppressed and renally compromised state. Surveillance data from France and other European countries suggest increasing incidence of non-cholera vibriosis, possibly related to climate change and rising coastal water temperatures (8, 9).

The bacteriological diagnosis of cholera and other *Vibrio* gastroenteritis relies on identification of the causative organism in stool specimens. *Vibrio* species are not fastidious in terms of culture: under optimal conditions, *V. cholerae* has a notably short generation time (approximately 10 min in liquid media), contributing to rapid colony appearance on agar plates. Most species grow well on blood agar and Hektoen enteric agar, although TCBS agar is preferred for primary isolation from stool because its alkaline pH and selective bile salts suppress competing enteric flora while allowing characteristic colony differentiation. On TCBS, sucrose-fermenting *V. cholerae* produces flat yellow colonies, whereas *V. parahaemolyticus* produces blue-green colonies (Table 1). Enrichment in alkaline peptone water (incubated at 35°C–37°C for 3–6 h) further enhances recovery but is not strictly necessary for most clinical samples. Chromogenic media, such as CHROMagar *Vibrio* and chromID *Vibrio*, provide an additional layer of presumptive species-level discrimination through colony color patterns, though confirmatory testing by MALDI-TOF remains required (10, 11).

While bacterial culture remains the gold standard for diagnosis of bacterial gastroenteritis, it is recognized that molecular diagnosis is more sensitive than culture. In 2010, Alam et al. highlighted limitations of the culture method for detection of *V. cholerae* among stool samples of cholera patients, including antibiotics, inactivation by *in vivo* vibriolytic action of a phage, or nonculturability induced as a host response (transformation of the bacteria into a coccoid nonculturable cell) (12). BD MAX is a multiplex molecular diagnosis panel providing rapid presumptive detection via the *atp*A gene, which is conserved across the *Vibrio* genus and present in all clinically relevant species, including *V. cholerae*, *V. parahaemolyticus*, and *V. vulnificus* (1, 13). Because this target does not allow species-level discrimination, a positive *atp*A result should always prompt culture, followed by MALDI-TOF identification. In non-endemic cholera settings, a positive *atp*A result may be dismissed as a false positive or attributed to more commonly expected species, such as *V. parahaemolyticus*, non-cholera *V. cholerae*, or exceptionally *V. vulnificus*. Microbiologists should be aware of which gene targets are included in their syndromic panels and which species carry those targets. Other molecular panels including *Vibrio* species are presented in Table 2.

**TABLE 2:** Main molecular diagnosis panels and their *Vibrio* gene target

Panels	Target	Encoding protein	Species
BD MAX Enteric Bacterial Panel (BD)	*atp*A	α-subunit of bacterial ATP synthase	*Vibrio* spp.
BioFire FilmArray GI (BioMérieux)	*gyr*B	ADN gyrase B	*V. parahaemolyticus*/*V. vulnificus* *V. cholerae* (cross-reactivity described for other *Vibrio* species)
	*tox*R	Transcriptional regulator gene	*V. cholerae*
Nanosphere’s Verigene Enteric Pathogens Nucleic Acid Test	*rfb*L	Tetronate biosynthesis (O antigen biosynthesis)	*V. cholerae*/*V. parahaemolyticus*
	*trk*H	Potassium transporter	
	*tna*A	Tryptophanase	
QIAstat-Dx Gastrointestinal Panel (QIAGEN)	Unpublished		*V. cholerae* *V. parahaemolyticus* *V. vulnificus*
Xpert GI panel (Cepheid)	Unpublished		*V. cholerae* *V. parahaemolyticus*
Allplex Gastrointestinal Bacterial plus Assay (Seegene)	Unpublished (one target per species)		*V. cholerae* *V. parahaemolyticus* *V. vulnificus*

MALDI-TOF spectrometry enables rapid and accurate identification of *Vibrio* species. Both the Bruker MALDI Biotyper and bioMérieux Vitek MS databases include *V. cholerae* reference spectra; the Bruker library used (MBT Compass HT IVD reference library version 2023; CE-IVD in Europe) contains entries for 55 *Vibrio* species. In our case, MALDI-TOF identification was achieved after less than 6 h of incubation on blood agar with a score of 2.45, which is above the 2.0 threshold for reliable species-level identification in the Bruker system. Nevertheless, species-level identification of *V. cholerae* by MALDI-TOF is not sufficient to confirm a cholera case. The species name “*cholerae*” is potentially misleading: only serogroups O1 or O139 strains carrying the *ctx*A gene and belonging to the seventh pandemic El Tor lineage (7PET) are responsible for cholera pandemic; non-toxigenic O1 strains also exist and do not cause cholera; as well as *V. cholerae* O1 *ctx*A positive strain that does not belong to the 7PET lineage and cause isolated cases or small outbreak linked to seafood consumption (14–16). Further characterization at a reference laboratory (including serogroup determination by slide agglutination and PCR detection of cholera toxin genes) is required to distinguish non-epidemic *V. cholerae* from epidemic cholera strains. Whole-genome sequencing is also recommended for *V. cholerae* O1 carrying *ctx*A gene in non-endemic cholera countries to confirm that the strain belongs to the 7PET lineage (17).

Regarding antibiotic susceptibility testing, EUCAST and CLSI provide specific clinical breakpoints for β-lactams, fluoroquinolones, tetracyclines, macrolides, and trimethoprim-sulfamethoxazole. However, agents to consider for primary testing are very different between these two recommendations, as MIC interpretative criteria are (see EUCAST Clinical Breakpoint Tables and CLSI M45 for more details). Antibiotics are usually not required in mild gastroenteritis; treatment is mainly based on rehydration. When antibiotic therapy is indicated, the 2017 IDSA guidelines recommend single-agent therapy (18). While there is no single standardized regimen for non-O1/non-O139 infections, French National Reference Center data (2018–2024) indicate that while resistance to ciprofloxacin (15.1%), trimethoprim-sulfamethoxazole (12.2%), ampicillin (8.8%), and tetracycline (7.9%) exists, resistance to azithromycin remains exceptionally rare (0%) (19). Thus, azithromycin appears to be a reasonable first-line choice, particularly to shorten symptom duration and reduce bacterial shedding.

The expanding use of syndromic PCR panels will likely increase detection of *Vibrio* species. Identification of *V. cholerae* by MALDI-TOF in a non-endemic cholera setting with no travel history to an endemic country or consumption of food or water from an endemic country (20) should prompt serogroup determination and toxin gene analysis rather than immediate alarm, as the majority of such isolates will be non-O1/non-O139 strains that do not cause epidemic cholera.

## SELF-ASSESSMENT QUESTIONS

1. Which of the following conditions is sufficient to confirm a case of cholera when a strain belonging to the species *V. cholerae* is isolated in stool specimens?
  - If identification is confirmed by MALDI-TOF spectrometry, the case of cholera is confirmed.
  - Serogroup confirmation and detection of toxin gene are not necessary.
  - Only strains of the species *V. cholerae* belonging to serogroups O1 and O139 and producing cholera toxin cause cases of cholera.
  - The isolation of a strain belonging to the species *V. cholerae* is sufficient to confirm a case of cholera from a patient with compatible clinical symptoms (acute watery diarrhea) in non-epidemic areas.
2. Which of the following statements regarding *Vibrio cholerae* identification is correct?
  - *V. cholerae* requires fastidious, specialized growth conditions and will not grow on standard non-selective media.
  - Enrichment in alkaline peptone water is strictly required for isolation of *V. cholerae* from clinical stool specimens.
  - TCBS agar is preferred for primary isolation of *V. cholerae* from stool because it suppresses competing enteric flora.
  - Chromogenic media, such as CHROMagar *Vibrio*, allow definitive species-level discrimination.
3. Which of the following statements concerning the treatment of non-O1/non-O139 *V. cholerae* gastroenteritis is correct?
  - Antibiotic treatment is always recommended, regardless of disease severity.
  - All strains of non-O1/non-O139 *V. cholerae* are susceptible to ciprofloxacin.
  - Azithromycin may be used as first-line antimicrobial therapy when treatment is indicated.
  - Antimicrobial susceptibility testing for *Vibrio* species uses the same interpretive breakpoints as for Enterobacterales.

## ANSWERS TO SELF-ASSESSMENT QUESTIONS

1. Which of the following conditions is sufficient to confirm a case of cholera when a strain belonging to the species *V. cholerae* is isolated in stool specimens?
  - If identification is confirmed by MALDI-TOF spectrometry, the case of cholera is confirmed.
  - Serogroup confirmation and detection of toxin gene are not necessary.
  - Only strains of the species *V. cholerae* belonging to serogroups O1 and O139 and producing cholera toxin cause cases of cholera.
  - The isolation of a strain belonging to the species *V. cholerae* is sufficient to confirm a case of cholera from a patient with compatible clinical symptoms (acute watery diarrhea) in non-epidemic areas.

Answer: (c) The isolation of a strain belonging to the species *V. cholerae* is not sufficient to confirm a case of cholera. Only strains of the species *V. cholerae* belonging to serogroups O1 and O139 that produce the cholera toxin and belong to the seventh pandemic lineage are responsible for cholera cases. Besides the fact that the clinical context must of course be taken into account, further investigations performed by a reference laboratory are necessary to distinguish non-choleric *V. cholerae* from cholera strains, including serogroup determination and detection of cholera toxin genes.

1. Which of the following statements regarding *Vibrio cholerae* identification is correct?
  - *V. cholerae* requires fastidious, specialized growth conditions and will not grow on standard non-selective media.
  - Enrichment in alkaline peptone water is strictly required for isolation of *V. cholerae* from clinical stool specimens.
  - TCBS agar is preferred for primary isolation of *V. cholerae* from stool because it suppresses competing enteric flora.
  - Chromogenic media, such as CHROMagar *Vibrio*, allow definitive species-level discrimination.

Answer: (c) *Vibrio* species are not considered fastidious organisms in terms of culture, and most grow well on non-selective media, such as blood agar and Hektoen enteric agar. Selective media, such as TCBS agar, are preferred for optimal isolation and differentiation, especially from mixed flora. Enrichment in alkaline peptone water can further enhance recovery but is not strictly necessary for most species in clinical samples. Chromogenic media may also be used and improve presumptive identification, particularly for *V. cholerae* and *V. parahaemolyticus*.

1. Which of the following statements concerning the treatment of non-O1/non-O139 *V. cholerae* gastroenteritis is correct?
  - Antibiotic therapy is always recommended, regardless of disease severity.
  - All strains of non-O1/non-O139 *V. cholerae* are susceptible to ciprofloxacin.
  - Azithromycin may be used as first-line antimicrobial therapy when treatment is indicated.
  - Antimicrobial susceptibility testing for *Vibrio* species uses the same interpretive breakpoints as for Enterobacterales.

Answer: (c) Antibiotics are usually not required in mild cases of gastroenteritis, and treatment is mainly based on rehydration. If antibiotic therapy is indicated, the 2017 IDSA guidelines recommend the use of a single-agent therapy. Resistance to ciprofloxacin is described (in approximately 15.1% of the strains), whereas azithromycin resistance remains exceptional. Antibiotic susceptibility testing can be performed by any laboratory using specific clinical breakpoints established by EUCAST or CLSI.

**TAKE-HOME POINTS**

- Isolation of *V. cholerae* from a clinical specimen does not confirm a cholera case. Only strains of the serogroup O1 or O139 carrying the *ctx*A cholera toxin gene and belonging to the El Tor seventh pandemic lineage are responsible for cholera.
- The *atp*A gene target of syndromic PCR panels is conserved across multiple *Vibrio* species and does not allow species-level discrimination. A positive result must always prompt culture and MALDI-TOF identification.
- Non-O1/non-O139 *V. cholerae* can cause gastroenteritis in immunosuppressed patients in non-endemic settings, even in the absence of travel or seafood exposure. The diagnosis should not be excluded on epidemiological grounds alone.
